# Maternal Antibodies Elicited by Immunization With an O- Polysaccharide Glycoconjugate Vaccine Protect Infant Mice Against Lethal *Salmonella* Typhimurium Infection

**DOI:** 10.3389/fimmu.2019.02124

**Published:** 2019-09-06

**Authors:** Scott M. Baliban, Brittany Curtis, Mohammed N. Amin, Myron Mike Levine, Marcela F. Pasetti, Raphael Simon

**Affiliations:** ^1^Center for Vaccine Development and Global Health, University of Maryland School of Medicine, Baltimore, MD, United States; ^2^Department of Medicine, University of Maryland School of Medicine, Baltimore, MD, United States; ^3^Department of Pediatrics, University of Maryland School of Medicine, Baltimore, MD, United States

**Keywords:** *Salmonella*, polysaccharide, flagellin, glycoconjugate, vaccine, antibody, maternal transfer, infant

## Abstract

Non-typhoidal *Salmonella* (NTS) are a leading cause of pediatric invasive bacterial infections in sub-Saharan Africa with high associated case fatality rates in children under 5 years old. We have developed glycoconjugate vaccines consisting of the lipid A-removed surface polysaccharide of NTS, core and O-polysaccharide (COPS), and the flagellar monomer protein (FliC) from the homologous serovar as the carrier. We previously established that COPS:FliC was immunogenic and protective in mice immunized as adults or infants; however, the brief period of murine infancy precluded the evaluation of protection against invasive NTS (iNTS) disease in early life. In the present study, we used a mouse model of maternal immunization to investigate transmission of *S*. Typhimurium COPS:FliC-induced maternal antibodies and protection against lethal iNTS challenge in infant mice. We found that vaccinated dams developed high levels of COPS- and FliC-specific IgG, which were transferred to their offspring. Sera from both vaccinated mothers and their litters mediated complement-dependent bactericidal activity *in-vitro*. Passively immunized 2-week old infant mice born to vaccinated mothers were fully protected from challenge with an *S*. Typhimurium blood isolate from sub-Saharan Africa. The pre-clinical findings reported herein demonstrate that anti-COPS:FliC antibodies induced by vaccination are sufficient for protection of murine infants against experimental *S*. Typhimurium infection. By underscoring the protective role of antibody, our results suggest that maintaining an adequate titer of protective anti-*Salmonella* antibodies during early life, either through pediatric or maternal COPS:FliC vaccination, may reduce iNTS disease in young children in sub-Saharan Africa.

## Introduction

Non-typhoidal *Salmonella* (NTS) are a leading cause of invasive bacterial infections in sub-Saharan Africa where children <5 years old bear the highest burden of disease with an estimated case fatality rate of ~20% (1–3). To date there are no approved human vaccines to prevent invasive NTS (iNTS) disease nor gastroenteritis. Blood culture surveillance of febrile children at multiple sites in sub-Saharan Africa has verified that pediatric iNTS infections occur largely within the first 30 months of life with the highest incidence amongst young children aged 1–2 years (4–7). Relative sparing of infants <5 months of age is seen, presumably due to passively acquired maternal antibodies. In support of this notion, an age cross-sectional study of Malawian children found that the decline in *Salmonella*-specific, complement-fixing antibodies in the sera of newborns to a minimum by 8 months of age correlated with the rise in iNTS disease that peaked around 12 months (6). Thereafter, serum bactericidal activity (SBA) rebounded with a concurrent decline in *S*. Typhimurium bloodstream infections. SBA titers in this study were strongly associated with the level of lipopolysaccharide (LPS)-binding antibodies. This finding is supported by other studies in humans and animal models that highlight a role for anti-LPS antibodies in mediating antibacterial activity against iNTS (8–10). Taken together, these observations suggest a putative protective role for maternal antibodies in preventing iNTS disease in young infants.

We previously reported the development of iNTS conjugate vaccines consisting of the core and O-polysaccharide (COPS) conjugated to the phase 1 flagellin protein (FliC) from the homologous serovar and established the immunogenicity and protective efficacy of COPS:FliC in mice (11–13). We have also demonstrated the immunogenic capacity of this vaccine in young mice (14). Given that murine IgG responses reach maturity at ~4–5 weeks of age (15), it was not feasible to assess protection during infancy after active immunization in our previous study, as multiple spaced doses are required prior to challenge, at which point all vaccine recipients would have reached adulthood (14). Another important unanswered question is whether COPS:FliC-induced antibodies are sufficient for protection of infant mice against iNTS infection. The goal of this study, therefore, was to determine if COPS:FliC-induced, maternally-transferred antibodies could provide protection against lethal invasive *S*. Typhimurium challenge during murine infancy.

## Materials and Methods

### Bacterial Strains

The bacterial strains used in this study are detailed in Table 1. Growth conditions for wild-type and reagent strains of *S*. Typhimurium were described previously (16).

**Table 1:** List of *S*. Typhimurium strains used in this study.

**Strain**	**Source/characteristics**	**References**
D65	Blood isolate, Mali, 2002	(7)
CVD 1925	*S*. Typhimurium I77 Δ*guaBA* Δ*clpP* Δ*fljB* Δ*fliD*	(16)
CVD 1925wzzB	*S*. Typhimurium I77 Δ*guaBA* Δ*clpP* Δ*fljB* Δ*fliD* (pSEC10-wzzB)	(13)

### Purification and Characterization of *S*. Typhimurium COPS and FliC

*S*. Typhimurium COPS and FliC were purified as described (13) from CVD 1925wzzB and isolate D65 to isolate I77, an attenuated derivative of the Malian blood isolate D65, which has deletions in *fljB* and *fliD* (lacks expression of phase II flagella and secretes phase I flagellar subunits), and overexpresses *wzzB*, ensuring the production of uniformly long-chain OPS (13, 17). Endotoxin removal was confirmed by endpoint limulus amebocyte lysate assay (Endosafe -PTS, Charles River, MA). Nucleic acid removal was confirmed by absorbance at 260 nm and Quant-iT dsDNA assay (Life Technologies, CA) for COPS and FliC, respectively. Removal of host cell protein in the COPS preparation was confirmed with the bicinchoninic acid (BCA) assay (Thermo, MA). COPS identity and relative molecular size were confirmed by Dionex HPAEC-PAD with commercially available monosaccharide standards and HPLC-SEC, respectively. FliC identification was accomplished by SDS-PAGE/Western blot with a monoclonal antibody specific for *S*. Typhimurium phase I flagellin [AH12IE6 (18)], and confirmation of monomeric form was accomplished by HPLC-SEC with detection at 280 nm.

### Synthesis of *S*. Typhimurium COPS:FliC Conjugates

COPS conjugates with FliC were synthesized as described (13). Briefly, COPS was derivatized at the reducing-end KDO residue with O-(3-mercaptopropyl)-hydroxylamine and linked to FliC, which had been derivatized at lysine amino groups with N-γ-maleimidobutyrl-oxysuccinimide ester (Molecular Biosciences, CO), using aminooxy-thioether chemistry. The conjugation reaction was fractionated by HPLC-SEC through Superdex 200 (GE, PA) to remove unconjugated components, and those fractions containing high molecular weight material were confirmed by SDS-PAGE/Coomassie staining with a PageRuler Plus molecular weight marker (Thermo). Fractions with conjugates ≥100 kDa were pooled for use in immunization experiments (13, 18).

### Ethics Statement

All animal studies were performed in facilities that are accredited by the Association for Assessment and Accreditation of Laboratory Animal Care. All animal experiments were in compliance with study protocols approved by the University of Maryland School of Medicine Institutional Animal Care and Use Committee. Experimental CD-1 adult females and male/female breeders were purchased from Charles River Laboratories (MA). Pups were bred and raised in the animal facility at the University of Maryland, Baltimore.

### Immunization, Sera Collection, and Challenge

Female CD-1 mice were immunized intramuscularly on days 0, 14, and 30 with COPS:FliC (2.5 μg polysaccharide) or PBS and were bred 35 days after the last dose. Blood was collected from dams 31 days post-vaccination (via retro-orbital sinus) and from pups 13–15 days post birth (via facial vein). Sera were stored at −20°C until analysis. For challenge studies, pups were infected intraperitoneally at 16–18 days of age with 5 × 10^2^ CFU of *S*. Typhimurium D65, which represents the minimum LD_100_ for 2 week old CD-1 mice. Challenged animals were monitored daily for signs of disease as described (13). Animals appearing moribund were euthanized and recorded as having succumbed to infection.

### ELISA

Serum anti-COPS IgG and anti-FliC IgG were detected by ELISA as previously described (13) and expressed as ELISA units (EU)/mL.

### Serum Bactericidal Activity

Serum bactericidal activity (SBA) was measured as previously described (13). Sera were heat-inactivated at 56°C for 30 min prior to use in the assay, and serially diluted in PBS. Fifty microliters of diluted antibody were mixed with 25 μL of baby rabbit complement (Pel-Freeze Biologicals, AR), 15 μL of PBS, and 10 μL of bacteria 100-350 CFU), incubated for 1 h at 37°C with mild shaking (115 rpm), and then plated to determine viable counts. Bacteria mixed with complement and PBS alone were used as negative controls. Individual SBA titers were determined as the highest dilution that produced > 50% reduction in CFU compared with controls containing buffer and complement alone, and the geometric mean titer (GMT) was then calculated. For COPS:FliC-immunized dams and litters, the SBA titers were normalized to a positive control (pooled *S*. Typhimurium D65 convalescent mouse sera generated in-house) and are representative of 2–3 independent experiments. PBS control mice were screened once.

### Statistical Analyses

Statistical analyses were performed using Prism v6 (GraphPad Software, CA), and no data points were excluded from analysis. For ELISA and SBA analyses, statistical comparisons were accomplished using a Mann-Whitney *U* test (two-tailed, α = 0.05). Correlations between maternal and pooled litter IgG and SBA titers were calculated using a Spearman correlation test (two-tailed, α = 0.05). Survival curves of challenged mice were compared by the log-rank test. Vaccine efficacy (VE) was calculated based on the attack rate (AR) in control and vaccinated mice as follows: VE = (AR_controls_-AR_vaccinated_)/AR_controls_) × 100. *P*-values ≤ 0.05 were considered to be statistically significant.

## Results

### Maternal Transfer of Anti-COPS IgG and Anti-FliC IgG From COPS:FliC-Immunized Dams to Their Offspring

**Figure 1:**
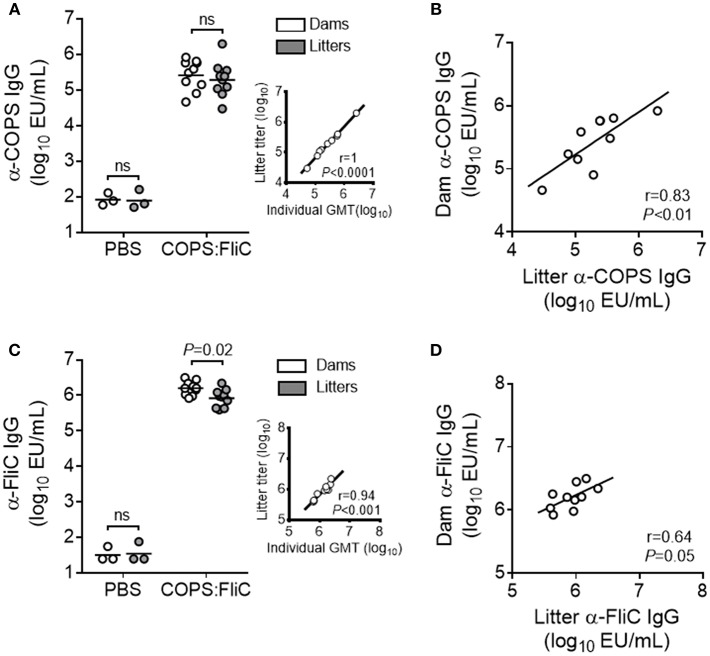
COPS:FliC-specific IgG antibody titers in the sera of mothers and their paired litters. Female CD-1 mice were immunized three times with PBS (*n* = 3) or COPS:FliC (*n* = 10) before breeding. Blood was collected from dams (d31 post vaccination) and their offspring (*n* = 11–15 pups/litter; d13-15 post birth). **(A)** Sera were screened for anti-COPS IgG titers in dams (white circles), their pooled litters (gray circles), or a random sampling of 7–9 pups from each litter (inset). The inset depicts a Spearman correlation between the 10 litter pool IgG titers and the pup IgG geometric mean titers (GMTs) for each litter (individual pup titers shown in Supplementary Figure 1). **(B)** Spearman correlations between dam and pooled litter IgG were calculated. Similar analyses were conducted for anti-FliC IgG titers **(C)** and paired maternal-litter IgG correlations **(D)**. In panels **(A,C)** (excluding insets), lines represent the GMT, and groups were compared using a Mann-Whitney *U* test. *P*-values ≤ 0.05 were considered to be significant. ns, not significant.

We previously demonstrated that immunization of female adult CD-1 mice with *S*. Typhimurium COPS:FliC conjugates induced robust titers of serum anti-COPS and anti-FliC IgG (13). To determine whether vaccine-specific antibodies could be passively transferred from COPS:FliC-immunized dams to their litters, female mice were vaccinated with COPS:FliC or PBS and bred 5 weeks following completion of the three dose regimen. Negligible titers of anti-COPS and anti-FliC IgG were found in the sera of PBS-immunized dams and their pups. In contrast, COPS:FliC-specific antibodies were readily detectable in vaccinated dams 1 month after the third dose and in their offspring 2 weeks after birth (Figures 1A,C). COPS:FliC-induced IgG titers from the litter pools were representative of the IgG GMTs for randomly sampled pups from each litter (Supplementary Figure 1). Maternal and infant serum anti-COPS IgG were significantly correlated (*r* = 0.83, *P* < 0.01) as well as anti-FliC IgG (*r* = 0.64, *P* = 0.05). Litters represented 32–240% and 24–101% of the titer from their paired mothers for anti-COPS IgG and anti-FliC IgG, respectively (Figures 1B,D).

### Functional Activity of Maternal COPS:FliC-Induced Antibodies in Pups

**Figure 2:**
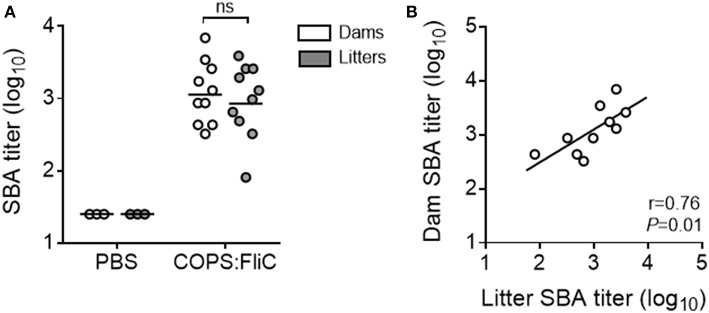
Complement-mediated bactericidal activity in the sera of PBS- and COPS:FliC-immunized mothers and their paired litters. Sera from groups of mice described in Figure 1 were assessed for serum bactericidal activity. Data represent titers from individual mothers (white circles) and their pooled litters (gray circles) **(A)**, and correlations between maternal and litter SBA titers were calculated using a Spearman correlation test **(B)**. In panel A, lines represent the GMT, and groups were compared using a Mann-Whitney *U* test. *P*-values ≤ 0.05 were considered to be significant. ns, not significant.

To address the functional capacity of COPS:FliC-specific antibodies, and whether maternally-acquired vaccine-induced antibodies retained their capacity to facilitate complement- dependent killing of *S*. Typhimurium, we measured SBA titers in the sera of dams and their paired litters (Figure 2A). As expected, sera from adult female mice had robust SBA against *S*. Typhimurium D65, a blood isolate from a febrile Malian child (GMT = 1,205). SBA was also seen in sera from offspring born to vaccinated dams, displaying a significant linear correlation with maternal SBA titers (19–200% of dam titer, *r* = 0.76, *P* = 0.01) (Figure 2B). By contrast, sera derived from PBS-control dams or their pups had no detectable killing of *S*. Typhimurium D65.

### Offspring of COPS:FliC-Immunized Dams Are Protected From Lethal *S*. Typhimurium Challenge

**Figure 3:**
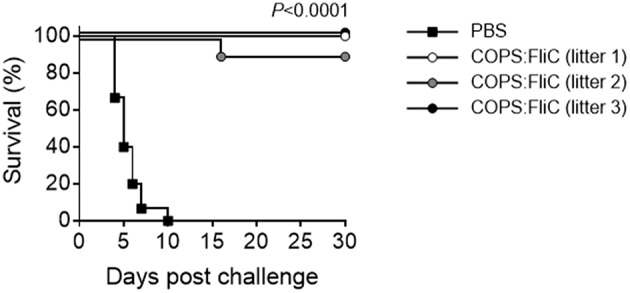
Infant mice born to COPS:FliC-immunized mothers are protected against lethal *S*. Typhimurium challenge. Litters from COPS:FliC-immunized mothers were challenged intraperitoneally with 5 × 10^2^ CFU of *S*. Typhimurium D65 (*n* = 11–15/group) 16 days post birth. Age-matched offspring from PBS-immunized females were used as a control. The Kaplan-Meier survival curves for individual COPS:FliC and PBS litters were compared using log-rank analysis. Corrections for multiple comparisons were not made. *P*-values ≤ 0.05 were considered to be significant. The survival curves are representative of two independent challenge experiments.

To assess whether maternally-transferred, COPS:FliC-induced antibodies would offer protection against iNTS challenge, 2 week-old pups born to PBS- or COPS:FliC-vaccinated mothers were infected intraperitoneally with 5 × 10^2^ CFU (MLD_100_ for 2-weeks of age) of virulent *S*. Typhimurium strain D65. All pups from PBS-treated mothers succumbed to challenge with *S*. Typhimurium D65 with a mean time to death of 5.5 days (Figure 3). In contrast, infant mice born to COPS:FliC-immunized dams were highly protected from lethal challenge, demonstrating vaccine efficacy of 91–100%. They did not exhibit any overt symptoms of disease, including ruffled fur, hunched posture, lethargy, or slowed growth.

## Discussion

Recent disease burden estimates obtained from 13 sites participating in the Typhoid Fever Surveillance in Africa Program (TSAP) indicated that children <5 years old bear the highest burden of iNTS bloodstream infections in sub-Saharan Africa (3), confirming previous estimates from systematic literature reviews (1, 2). Bactericidal antibodies recognizing the O-polysaccharide of iNTS are detectable in newborns, and their levels decline over the first year of life, commensurate with a rise in iNTS bacteremia (6). Pediatric iNTS vaccines which elicit humoral immune responses against the O-polysaccharide moiety are thus warranted; however, newborns and young infants typically respond poorly to isolated carbohydrate molecules. Glycoconjugate vaccines overcome this limitation and the utility of this approach in the African setting is exemplified by the reduction in invasive bacterial disease in children caused by *Haemophilus influenzae* type B (Hib) following programmatic introduction of Hib conjugate vaccines into the Malian Expanded Programme on Immunization (EPI) (19). The capacity of iNTS-specific antibodies to confer protection in early life has been demonstrated in animal models where the offspring of mothers immunized with either live attenuated or inactivated whole cell NTS vaccines were protected from experimental iNTS infection (20–22). It was unknown however whether humoral immunity induced by an OPS-based glycoconjugate vaccine would similarly offer protection to infant mice. Here, we demonstrated that COPS:FliC-induced antibodies and SBA against *S*. Typhimurium are efficiently transferred from mother to offspring and confer robust protection of murine infants against lethal challenge with a clinically relevant strain of *S*. Typhimurium from sub-Saharan Africa. This study, to our knowledge, is the first to evaluate the efficacy of COPS-based glycoconjugate-induced antibodies for protection of infant mice against lethal iNTS infection using a model of maternal vaccination and passive transfer.

Rodents can vertically transmit IgG through both the placenta *in utero* and breast milk after birth, potentially reaching serum levels similar to or higher than that of the dam (23). Concordantly, maternal-derived COPS- and FliC-specific IgG were readily detectable in the sera of 2-week-old pups born to COPS:FliC-immunized dams. Sera from these immune litters demonstrated robust bactericidal activity, which approached that of their respective dams. Accumulating *in-vitro* evidence suggests that antibodies against *S*. Typhimurium LPS and FliC can independently mediate complement-dependent killing (24–26); therefore, both anti-polysaccharide and anti-protein antibodies likely contribute to SBA in our model. *S*. Typhimurium is also sensitive to killing via antibody-mediated bacterial phagocytosis followed by oxidative burst (27). Serum antibodies from *S*. Typhimurium COPS:FliC-vaccinated adult mice are robustly opsonophagocytic (13). Hence, we expect this antibody functionality is also transferred to the offspring of COPS:FliC-immunized mothers, although this remains to be confirmed. Remarkably, maternal antibody was sufficient to provide near full protection against lethal *S*. Typhimurium challenge in 2-week old pups, regardless of the heterogeneity in transfer efficiency for either COPS:FliC-specific IgG or SBA. These results are in agreement with our previous findings in adult mice whereby passive transfer of COPS:FliC-induced mouse or rabbit antisera provided robust protection against iNTS challenge (11, 28).

Maternal COPS:FliC-specific antibodies were efficiently transferred to litters, although some heterogeneity was noticed when comparing SBA transfer rates. This observation may in part result from the genetic diversity among outbred CD-1 mice or variations in maternal milk production and consumption. It may also reflect intrinsic differences in maternally transferred antibodies, such as IgG subclass and Fc glycan patterns, which have been documented to vary between matched maternal serum and cord blood in humans (29). The putative influence of selective maternal transfer on antibody-mediated killing of iNTS will be the focus of future studies. A caveat to our SBA protocol is that dam and litter sera were compared using an equivalent amount of exogenous complement. In humans, the systemic concentration of complement proteins in neonates is lower than adults, gradually rising to mature levels over the course of 12–18 months (30). The SBA assay may therefore overestimate the bactericidal activity of maternally-acquired antibody within infant mice. If infant complement levels were limiting such that membrane attack complex formation and bacterial lysis were inefficient during challenge, other antibody effector mechanisms (e.g., opsonophagocytic killing or antibody dependent cell cytotoxicity), may become more important for protection. Future studies should also address the relative contribution of different antibody functionalities for protection against iNTS early in life.

The pre-clinical findings reported herein have potentially important translational implications as these data suggest that attainment of a critical level of COPS:FliC-induced antibodies may offer protection during early life. Prior to vaccination, young human infants in endemic areas may already have an appreciable titer of maternally-derived anti-*Salmonella* antibodies due to natural exposure of their mothers. In humans, maternally-inherited antibodies can persist for several weeks to months after birth and have been reported to interfere with adequate seroconversion in infants to live and subunit vaccines, thus blunting vaccine-mediated protection in a manner dependent upon the titer of maternal antibody (31, 32). Prior clinical studies conducted in Vietnam with a Vi capsular polysaccharide-based typhoid conjugate vaccine found that higher levels of anti-Vi IgG in cord blood taken from mothers directly after birth correlated with diminished anti-Vi antibody responses in their vaccinated infants (33). It is unknown, however, whether maternally-transferred COPS- or FliC-specific antibodies would affect the priming of infant immune responses by COPS:FliC conjugates. Additional pre-clinical studies are needed to address the impact of maternal iNTS-specific antibodies on active COPS:FliC immunization. Nevertheless, our data highlight the sufficiency of anti-COPS and anti-FliC antibodies for protection of murine infants against experimental iNTS challenge and provide important pre-clinical proof-of-concept for an NTS glycoconjugate vaccine that may confer protection against pediatric iNTS disease in sub-Saharan Africa upon incorporation into the African EPI.

## Ethics Statement

All animal studies were performed in facilities that are accredited by the Association for Assessment and Accreditation of Laboratory Animal Care. All animal experiments were in compliance with study protocols approved by the University of Maryland School of Medicine Institutional Animal Care and Use Committee. Experimental CD-1 adult females and male/female breeders were purchased from Charles River Laboratories (MA). Pups were bred and raised in the animal facility at the University of Maryland, Baltimore.

## Author Contributions

RS and MP conceived and designed the experiments. RS acquired funds for the study and was responsible for project administration. SB, MA, and BC performed the experiments. SB, MP, and RS formally analyzed the data. SB, ML, MP, and RS wrote and edited the paper.

### Conflict of Interest Statement

RS and ML are co-inventors on patents describing *Salmonella* COPS:FliC conjugate vaccines. The funding sponsor had no role in the design of the study; in the collection, analyses, or interpretation of data; in the writing of the manuscript, and in the decision to publish the results. The remaining authors declare that the research was conducted in the absence of any commercial or financial relationships that could be construed as a potential conflict of interest.

## Supplementary Material

Supplementary Figure 1 Anti-COPS IgG and anti-FliC IgG titers amongst individual pups born to COPS:FliC-vaccinated dams. Pups from COPS:FliC-immunized mothers (n = 7-9 pups/litter, described in Figure 1) were screened for serum IgG titers against COPS **(A)** and FliC **(B)**. In both panels, litters are placed in ascending order according to the anti-COPS IgG titer. Points represent individual mice, and lines indicate the geometric mean titer (GMT).
