# Supplementary figures and images for: Maternal Antibodies Elicited by Immunization With an O- Polysaccharide Glycoconjugate Vaccine Protect Infant Mice Against Lethal Salmonella Typhimurium Infection
Source: Front Immunol. 2019 Sep 6;10:2124. doi: 10.3389/fimmu.2019.02124 (PMC6743215; doi:10.3389/fimmu.2019.02124)

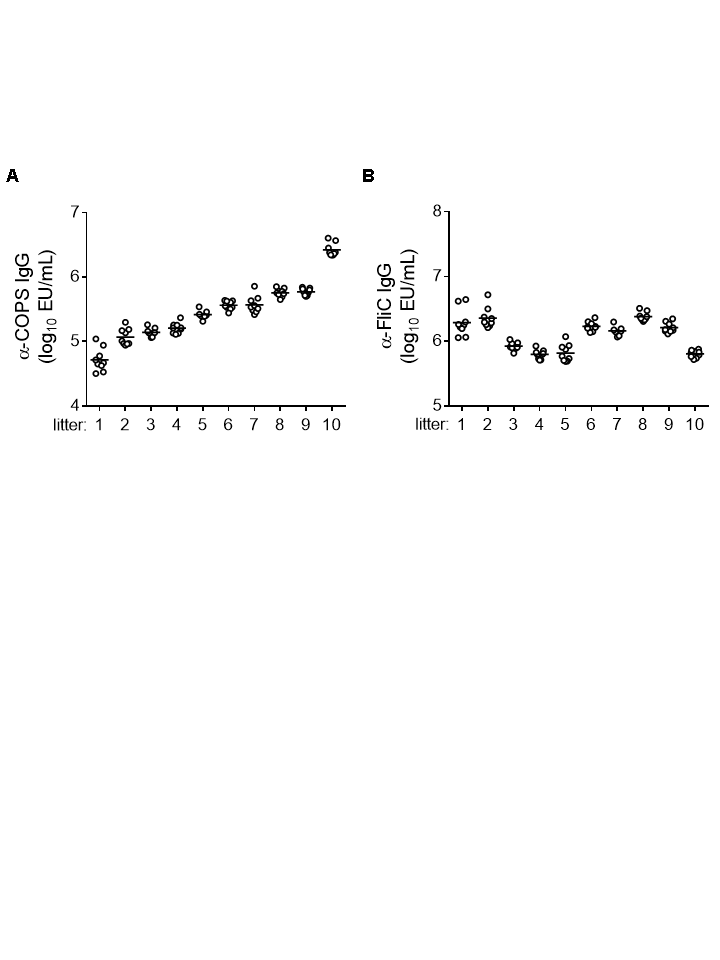

Supplement: Supplementary Figure 1 — Anti-COPS IgG and anti-FliC IgG titers amongst individual pups born to COPS:FliC-vaccinated dams. Pups from COPS:FliC-immunized mothers (n = 7-9 pups/litter, described in Figure 1) were screened for serum IgG titers against COPS (A) and FliC (B). In both panels, litters are placed in ascending order according to the anti-COPS IgG titer. Points represent individual mice, and lines indicate the geometric mean titer (GMT). [file Image_1.TIF]
