# Supplementary material for: Maternal Antibodies Elicited by Immunization With an O- Polysaccharide Glycoconjugate Vaccine Protect Infant Mice Against Lethal Salmonella Typhimurium Infection
Source: Front Immunol. 2019 Sep 6;10:2124. doi: 10.3389/fimmu.2019.02124 (PMC6743215; doi:10.3389/fimmu.2019.02124)
Supplement: Supplementary file 2 [file Table_1.DOCX]

Supplementary Material

# Supplementary Figures

## Supplementary Figure 1. Anti-COPS IgG and anti-FliC IgG titers amongst individual pups born to COPS:FliC-vaccinated dams.

Pups from COPS:FliC-immunized mothers (*n* = 7-9 pups/litter, described in Figure 1) were screened for serum IgG titers against COPS **(A)** and FliC **(B)**. In both panels, litters are placed in ascending order according to the anti-COPS IgG titer. Points represent individual mice, and lines indicate the geometric mean titer (GMT).

**2 References**

1. Tapia MD, Tennant SM, Bornstein K, Onwuchekwa U, Tamboura B, Maiga A, et al. Invasive Nontyphoidal Salmonella Infections Among Children in Mali, 2002-2014: Microbiological and Epidemiologic Features Guide Vaccine Development. Clin Infect Dis. 2015;61 Suppl 4:S332-8.

2. Tennant SM, Wang JY, Galen JE, Simon R, Pasetti MF, Gat O, et al. Engineering and preclinical evaluation of attenuated nontyphoidal Salmonella strains serving as live oral vaccines and as reagent strains. Infect Immun. 2011;79(10):4175-85.

3. Baliban SM, Yang M, Ramachandran G, Curtis B, Shridhar S, Laufer RS, et al. Development of a glycoconjugate vaccine to prevent invasive Salmonella Typhimurium infections in sub-Saharan Africa. PLoS Negl Trop Dis. 2017;11(4):e0005493.
